# Effect of Fiber Material on Tribological Performance of Filament-Winding Composite Materials in a Water-Lubricated Environment

**DOI:** 10.3390/polym18020269

**Published:** 2026-01-19

**Authors:** Yicong Yu, Zhijun Chen, Zhiwei Guo

**Affiliations:** 1 School of Transportation and Logistics Engineering, Wuhan University of Technology, Wuhan 430063, China; yicongyu@whut.edu.cn; 2 State Key Laboratory of Waterway Traffic Control and Safety, Wuhan University of Technology, Wuhan 430063, China; 3 Reliability Engineering Institute, National Engineering Research Center for Water Transportation Safety, Wuhan 430063, China; 4 School of Naval Architecture, Ocean and Energy Power Engineering, Wuhan University of Technology, Wuhan 430063, China

**Keywords:** water-lubricated bearing, filament-winding, tribological performance, filament-winding composite materials

## Abstract

Water-lubricated bearings are critical components in marine propulsion systems, necessitating materials with exceptional tribological properties to ensure reliability. Filament-winding technology is an effective molding method for enhancing the comprehensive properties of polymers, and the selection of fiber materials has a significant impact on the performance of polymers. In this study, three types of polyurethane (PU) matrix filament-winding composites were fabricated via filament-winding technology. Under water-lubricated conditions, a friction test (disk-to-disk) with a duration of 2 h was performed, followed by systematic observations of the resultant wear behavior. The results indicate that aramid fibers exhibited the superior reinforcing effect on the PU matrix, effectively suppressing wear while enhancing mechanical properties. Specifically, under the conditions of 0.5 MPa-250 r/min (0.314 m/s), the minimum friction coefficient of the aramid fiber-wound composite material was 0.093, which was 57.73% lower than that of pure polyurethane. Under the conditions of 0.7 MPa-50 r/min (0.0628 m/s), the wear mass of the sample was limited to only 1.5 mg, which was 12% lower than that of polyurethane. This research can provide a practical reference for the application of filament-wound composite materials in water-lubricated bearings.

## 1. Introduction

The stern bearing is a key component of the ship’s shafting system, supporting the rotation of the stern shaft and maintaining operational stability [1,2]. Water-lubricated bearings, as a green alternative, have advantages, such as environmental protection and resource conservation, and are widely used in maritime transportation. However, compared with oil lubrication, water medium has a lower viscosity and is prone to form an unstable thin water film under high-load conditions, resulting in a poor lubrication state and intensifying the friction and wear of water-lubricated bearings [3]. Therefore, further research on material optimization and lubrication mechanisms is needed to enhance the reliability and service life of these particular bearings.

Fiber-reinforced composites are widely used in multiple fields such as aerospace, construction, and automobiles due to their excellent performance, like high strength, lightweight, and corrosion resistance [4,5,6]. Modifying the matrix material with fibers has become an effective method to improve the mechanical and tribological performance of polymers. Fiber-reinforced composites combine different fibers and matrix materials, and their performance is superior to that of pure metals, polymers, or alloys, such as increased strength and stiffness, reduced weight, as well as impact resistance, wear resistance, corrosion resistance, and chemical resistance [7]. Kunishima et al. [8] studied the tribological performance of carbon/glass fiber reinforced polyamide (PA66) matrix composites. The results showed that the glass fiber reinforced PA66 composites exhibited better tribological performance under grease lubrication, while the carbon fiber composites showed a lower coefficient of friction under dry conditions. Yu et al. [9] developed an organic material brake pad with high friction stability using basalt fiber. The research results showed that the use of basalt fiber could enhance the mechanical properties, thermal properties, and friction coefficient of the braking material and also improve the friction stability. The friction stability mechanism of basalt fiber was explained in detail at both the macroscopic and microscopic levels.

Fiber-winding-forming technology has become highly mature. It refers to the continuous winding of resin-impregnated fibers or pre-impregnated tapes onto a core mold or die under a given tension, at a preset angle and line shape, and then curing and forming at room temperature or high temperature [10]. Subsequently, the formed components undergo post-treatment operations, such as demolding and polishing, to ultimately obtain composite material products that meet the requirements. İpekci et al. [11] discovered that the filament-winding-forming method using 6-axis robot additive manufacturing technology can produce composite material elbows with excellent mechanical properties without the use of molds. This method significantly enhances the internal pressure strength of the product by optimizing production parameters, demonstrating the advantages of winding-forming in the manufacturing of complex structures.

For fiber-wound composites, the selection of different fibers under the same matrix material will have a significant impact on the performance of the composites. Wang et al. [12] conducted a tribological analysis on the short fiber-reinforced ultra-high molecular weight polyethylene (UHMWPE) composite material formed by hot pressing and sintering under water lubrication and dry friction conditions and found that the characteristics of different fiber materials have different influences on the tribological performance of the composites. Fatemeh et al. [13] conducted a study on the tribological and mechanical properties of the wound composite material containing polytetrafluoroethylene (PTFE) fibers and tungsten carbide fillers in self-lubricating bearings. Research on composite materials prepared from different fibers has revealed that the content of PTFE has a crucial influence on the performance of the composite material.

Apart from inorganic fibers, such as carbon fiber and glass fiber, organic fibers have also played a significant role in fiber-reinforced composite materials in recent years. Synthetic organic fibers such as aramid fibers, UHMWPE fibers, and polypropylene fibers have shown great application potential in the field of water-lubricated bearings due to their excellent mechanical properties, acid and alkali resistance, and wear resistance. Eom et al. [14] conducted a study on short aramid fiber-reinforced thermotropic liquid crystalline polyester composites and found that the introduction of short aramid fibers (AFs) could effectively improve the melt rheological behavior, thermal transition characteristics, thermal stability, and mechanical durability of the composite materials. Additionally, the addition of 5 wt% AF significantly increased the elastic modulus and the long-term mechanical durability at high temperatures. Wang et al. [15] conducted studies on the molecular chain disentanglement and crystallization characteristics of spinning and fibers, as well as the influence of structure on the final mechanical properties of fibers by adding different amounts of high-molecular-weight polyethylene (HDPE). The results showed that increasing the content of HDPE in the UHMWPE/HDPE blended fibers could improve the melt processing properties, and due to the compact crystal structure and fully stretched molecular chains, better mechanical properties were obtained. Huang et al. [3] prepared a composite material similar to Lignum vitae using hollow polypropylene fibers (PPFs) and thermosetting polyurethane. The research results showed that this material exhibits excellent tribological properties under both water lubrication and dry friction conditions. The filament-winding angle has a significant effect on the tribological performance of the composite. Among which, a winding angle of 55° shows the best capacity of load distribution and advantages in lubrication secretion.

In this study, three organic fibers (aramid fiber, ultra-high molecular weight polyethylene fiber, and polypropylene fiber) were selected as reinforcing phases, and thermosetting polyurethane was used as the matrix to prepare fiber-wound composites. Friction and wear tests were conducted under different test conditions. The tribological performance of composite materials is comprehensively evaluated by analyzing parameters such as mechanical properties, friction coefficient, and wear mass loss. Combined with the microscopic morphology of the material surface after wear, the wear state and process are analyzed to explore the influence of fiber material on the tribological performance of fiber-wound composite materials. This research provides valuable guidance for the material selection and structural optimization of water-lubricated bearings operating under harsh conditions.

## 2. Materials and Methodology

### 2.1. Material Preparation

The original materials used for filament winding are polyurethane (PU) (Nanjing Jufa New Material, Co., Ltd., Nanjing, China) and three types of organic fibers (Jiangxi Shengli Special Materials Technology Co., Ltd., Xinyu, China), which are para-aramid fiber, ultra-high molecular weight polyethylene fiber, and polypropylene fiber, respectively. The densities of the three kinds of fibers are all 1000D. Based on the analysis of the molecular mass and properties of the raw materials, PU is a mixture of polyether polyols (Urewin2301) and isocyanates (Urewin2307), with a mass ratio of 100 g:46 g. After thoroughly mixing polyether polyols and isocyanates at room temperature (25 °C), the resulting polyurethane pre-polymer cures slowly and can maintain a low viscosity for more than 4 h at room temperature to adapt to the filament-winding process.

### 2.2. Filament-Winding Process

**Figure 1:**
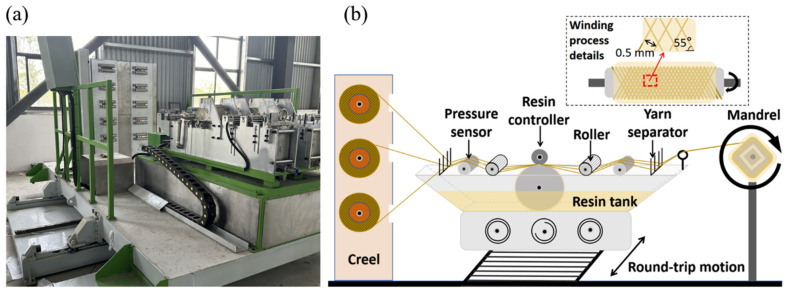
CNC filament-winding machine: (**a**) physical picture; (**b**) schematic diagram.

In this study, the computer numerical control (CNC) filament-winding machine (DQ 003) (Zhongtong Composite Materials Machinery Equipment Manufacturing Factory, Lianyungang, China) was used for material preparation. The schematic diagram of the CNC filament-winding machine is shown in Figure 1. The main components include the creel, damper, rail-guided vehicle, resin tank, pressure sensor, resin controller, roller, yarn separator, mandrel, and the motor responsible for providing power to the system.

The mandrel is a component that receives the fiber bundles through rotation during the fiber-winding process. In order to ensure that the wound fiber bundles are consistent with the movement direction during the friction process and considering the processability, a square mandrel was adopted in this study. The resin tank is the location where the resin is stored in the fiber-winding machine. A large cylindrical roller was kept rolling, coating its side surface with resin. At the same time, a small cylindrical resin scraper was fixed above the roller. The two cylinders were in rolling contact. The amount of resin impregnated in the fiber bundle was controlled by regulating the gap between the two cylinders. Pressure sensors were installed both before and after the resin controller, which can display the tension of the fiber bundle. The initial tension of the fiber bundle was controlled by adjusting the tightness of the damper on the creel [16]. Before the test began, the wound fibers were placed in an oven at 80 °C ± 2 °C for drying for 8 h to remove the original moisture on the surface of the fibers. After drying, the fiber rolls were placed on the creel, and then the fiber filaments were pulled out for wiring. The fiber filaments were passed through the pressure sensor, the resin controller, the roller, and the yarn separator in sequence and were finally fixed on the mandrel. The process parameters were set, such as the winding angle, winding speed, winding tension, and the number of winding layers. Finally, the resin solution was poured into the resin tank, the motor was turned on to make the mandrel start to rotate, and at the same time, the rail-guided vehicle began to move back and forth along the track to start preparing the fiber-wound composite material sample.

During the winding process, the angle between the fiber bundle and the mandrel affects the mechanical properties of the fiber-winding material. According to published research, the optimal winding angle of the fibers is between 40° and 70° [17,18]. To keep the variables constant, the process parameters of numerical control winding remain consistent. The winding angle was 55°, the winding speed was 55 r/min, and the winding tension was controlled within 12–15 N. When the thickness of the fiber composite material on the core mold reached 15 mm, the winding process was completed. The core mold and the fiber composite material were removed together and placed in the curing furnace. They were cured according to the curing procedure of 1 h/100 °C + 1 h/120 °C. To ensure uniform heating, the samples in the curing furnace were heated by rotating at a constant speed. After cooling to room temperature, the curing furnace was turned on. The samples were taken out and demolded with a demolding machine to obtain the fiber-wound composite material samples. We named the polyurethane samples wound with three different fibers (aramid fiber, ultra-high molecular weight polyethylene fiber, and polypropylene fiber) ARF, UF, and PPF, respectively.

### 2.3. Material Characterization Techniques

The mechanical properties of the material, such as tensile strength, compressive strength, and flexural strength, were measured by using the universal material testing machine (Instron 5967, Co, Ltd., Norwood, MA, USA) in accordance with the test standards GB/T 2567-2021 (PU) [19], GB/T1040.1-2025 [20], GB/T1448-2005 [21], and GB/T1449-2005 [22]. Shore hardness of samples was tested by a Shore hardness tester (HLX-AC, Adelberg Handpi Instrument Co., Ltd., Yueqing, China). The surface morphology of the polymer disk was observed by a scanning electron microscope (VEGA3, Tescan Co., Ltd., Brno, Czech Republic). Before scanning electron microscopy (SEM) measurements, all samples were gold-plated by sputtering. A laser scanning confocal microscope (VK-X2000, Keyence Ltd., Osak, Japan) was used to observe the wear surface of the grinding pair.

### 2.4. Tribological Test

All the tribological tests were conducted on a CBZ-1 friction and wear testing machine (Haima Co., Ltd., Haikou, China). QSn7-0.2 is a commonly used material for bushings in ship shafting systems [23]. Therefore, the friction pair in this study was composed of a tin bronze plate (90~92% Cu, 0.3% Zn, 6~8% Sn, 0.2% Ni, 0.01% Al, 0.02% Pb, 0.15% others) and fiber-wound composite material samples. The mechanical properties of the tin bronze plate are shown in Table 1. In order to ensure that the friction pair maintains full-area contact and friction stability in tribological tests, the outer diameter of the tin bronze plate surface was slightly larger than that of the sample, and the inner diameter was slightly smaller than that of the sample [24]. The outer diameter of the composite material sample was 30 mm, the inner diameter was 18 mm, and the height was 10 mm. The outer diameter of the tin bronze plate was 32 mm, the inner diameter was 16 mm, and the height was 6 mm.

**Figure 2:**
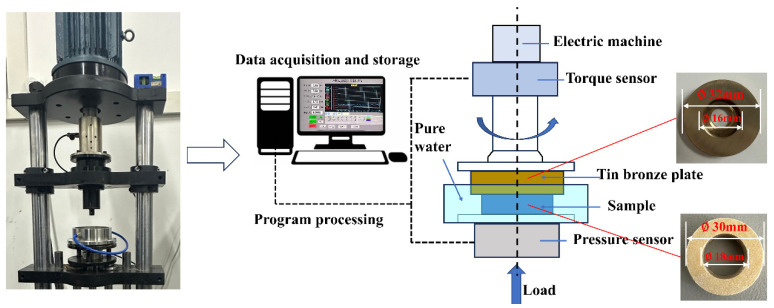
Schematic diagram of tribological test.

Consistent with previous studies [25,26,27], the test samples were fixed on the base of the testing machine and completely immersed in pure water. The tin bronze grinding piece was driven by a motor to slide on the surface of the sample, and the load and speed of the test were adjusted through the control device of the friction and wear testing machine, allowing the sample’s circular ring to conduct a disk-to-disk grinding test with the tin bronze plate. The schematic diagram of the tribological test is shown in Figure 2.

The test conditions were selected in accordance with the military standard MIL-DTL-17901 of NAVSEA (SH) [28,29]. The rotational speeds of the test spindle were set at 50 r/min, 150 r/min, and 250 r/min to simulate the working conditions of the ship at low speed and in normal operation. The loads applied to the friction pair were set at 0.5 MPa and 0.7 MPa, respectively, to simulate the working conditions under normal load and heavy load so as to explore the tribological performance of the composite material under normal and relatively harsh working conditions [30]. To ensure the reliability of the test results, the test was repeated three times under each friction and wear test condition to eliminate random errors.

Before the test, the surfaces of the tin bronze plate and the resin matrix samples were polished with 240, 600, and 1000 grit sandpapers on the polishing machine to make the surface roughness of the friction pairs the same. The average surface roughness, Ra, was approximately 0.8 μm. A measurement length of 2000 μm was selected along the circumference of the ring. The polished material was rinsed with distilled water, dried in an oven for 12 h, and then weighed. The average quality was calculated after conducting three quality tests. In the experiment, when changing materials or altering working conditions, the water tank was cleaned with distilled water, and continuous grinding was carried out for 7200 s under each working condition. After the test was completed, the tin bronze plate and the resin sample were washed, dried, and weighed again. By comparing the sample quality before and after the test, the wear data of the sample during the friction and wear test can be calculated.

## 3. Results

### 3.1. Water Absorption

Considering that the addition of organic fibers usually causes significant swelling of the material in water, it may affect the application of the composite material as a water-lubricated bearing. According to the US military standard MIL-DTL-17901 of NAVSEA (SH), for water-lubricated bearings made of plastic material as a substrate, the change in volume of the sample made of this material after soaking in water for 7 days should be less than 5%, and if the volume increase is less than 1%, it is grade IV, and if it is less than 0.2%, it is grade V [31].

To evaluate the differences in water absorption of different fiber-wound composite samples, samples of ARF, UF, PPF, and pure PU with dimensions of 30 mm × 10 mm × 10 mm were immersed in distilled water at 20 °C in a 1 L beaker. Measure and record the average dimensional changes in the sample at different soaking times with a vernier caliper, and calculate the average volume growth rate of the sample at different soaking times. Table 2 shows the average volume changes in samples soaked in distilled water for different times.

The test results show that the addition of the three organic fibers all increases the swelling property of the polyurethane matrix. However, the swelling properties of the three composites, ARF, PPF, and UF, all meet the standards. Among them, the swelling property of ARF is smaller compared with the other two fiber-wound composites.

### 3.2. Mechanical Properties

**Figure 3:**
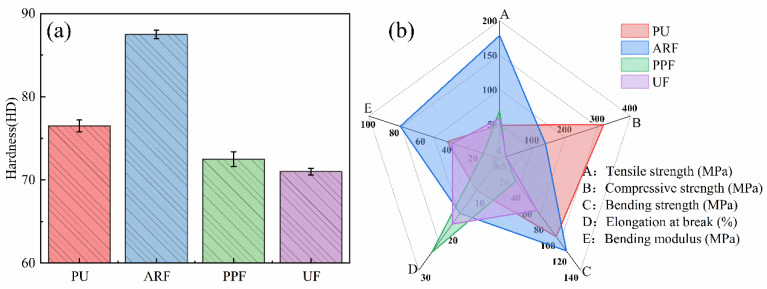
Mechanical properties of different filament-winding composite materials: (**a**) Hardness; (**b**) Strength and deformation performance.

The mechanical properties of materials are directly related to their reliability and durability in practical use. When studying the tribological and lubricating properties of materials, it is of great significance to study their mechanical properties. Therefore, in this study, tensile, compressive, and bending strengths and hardness tests were conducted on four different material samples, and the test results are shown in Figure 3.

The analysis of the test data on mechanical properties reveals that the tensile strength of the three composite materials, ARF, PPF, and UF, has increased to varying degrees compared with PU. This is because the fibers, as reinforcing phases, preferentially bear the load during the tensile process and effectively share the stress of the matrix. Among them, the tensile strength of ARF is much greater than that of the other three composite materials, reaching 178.5 MPa, which is 3.7 times that of PU. This indicates that aramid fibers have the characteristics of high modulus and high strength. In the PU matrix, they can effectively bear external forces and disperse stress, thereby greatly enhancing the tensile strength of the material.

The compressive strengths of ARF, PPF, and UF all decreased to varying degrees compared with PU (318 MPa). This is related to the fact that fibers with a high aspect ratio are prone to micro-buckling under compressive loads, resulting in local stress concentration and weakening the compressive resistance. The bending strengths of the four samples also vary significantly. Aramid fibers increase the bending strength, while the bending strength of materials reinforced with polypropylene fibers and UHMWPE fibers is lower than that of PU. This indicates that aramid fibers and PU have a good synergistic effect. When subjected to bending loads, they can prevent the propagation of cracks and enhance the bending strength of the material. This is consistent with the calculated bending modulus result of the specimens. By comparing the Shore hardness of the materials, it can be found that the addition of aramid fibers significantly increases the hardness of the composite material to 87.5 HD, which is 14.4% higher than that of PU. However, the addition of polypropylene fibers and UHMWPE fibers instead makes the hardness of the composite material lower than that of PU. This is related to the characteristics of the fiber material. The research result that PPF and UF have relatively larger elongation at break also indicates this point. Aramid fibers with relatively high hardness can enhance the entire composite material’s resistance to local deformation, thereby increasing its hardness. In contrast, the addition of flexible fibers such as polypropylene fibers and UHMWPE fibers reduces the overall rigidity of the material.

Overall, ARF demonstrates significant advantages in tensile strength, flexural strength, and hardness, but its compressive strength is slightly lower than that of PU. PPF and UF outperform PU in tensile strength, but they have different performances in compressive strength, flexural strength, and hardness. From this, it can be seen that different fiber materials have a very significant influence on the mechanical properties of fiber-wound composites.

### 3.3. Friction Coefficient Analysis

**Figure 4:**
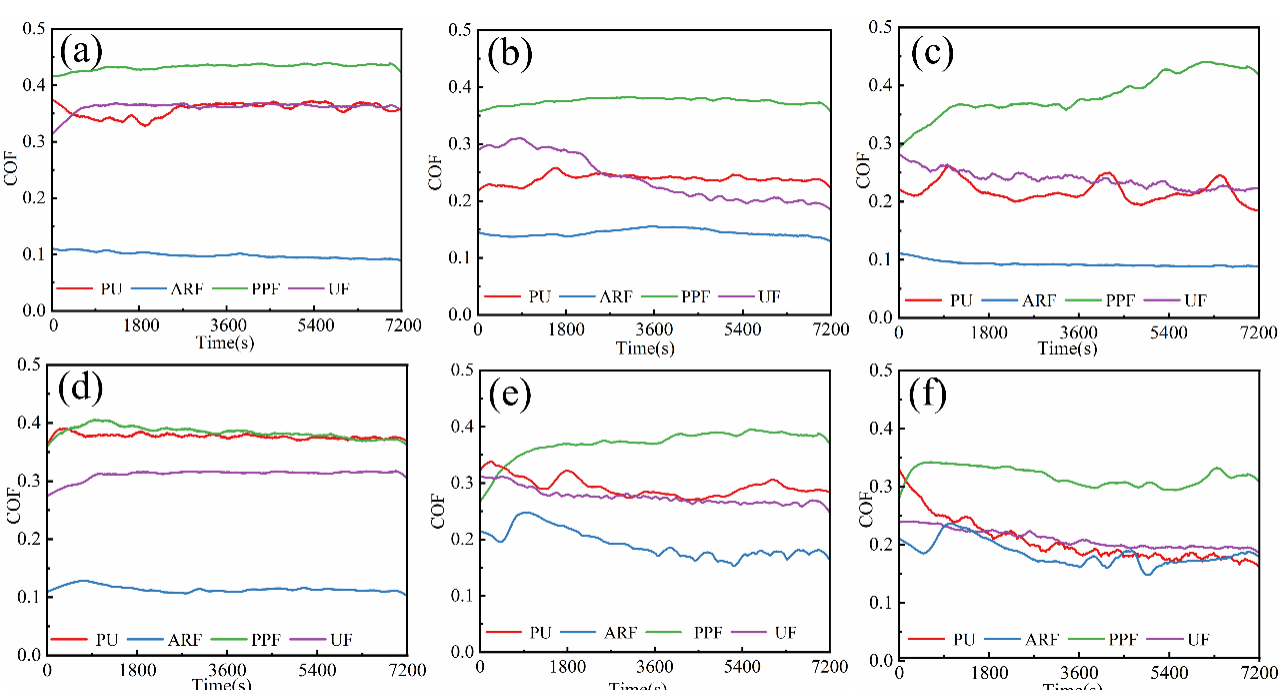
Real-time COF of materials under different test conditions: (**a**) 0.5 MPa-50 r/min; (**b**) 0.5 MPa-150 r/min; (**c**) 0.5 MPa-250 r/min; (**d**) 0.7 MPa-50 r/min; (**e**) 0.7 MPa-150 r/min; and (**f**) 0.7 MPa-250 r/min.

**Figure 5:**
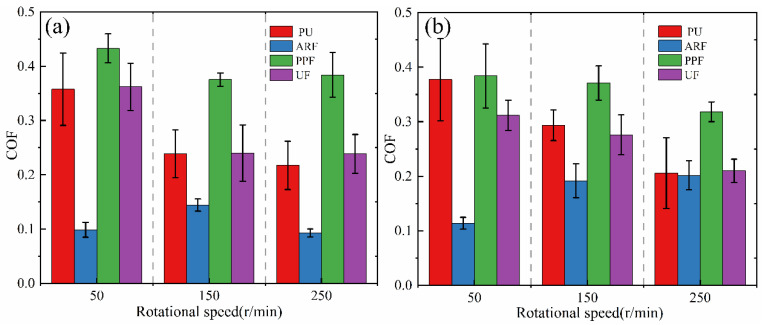
The average COF of the samples under different loads (the error bars represent the standard deviation): (**a**) 0.5 MPa; (**b**) 0.7 MPa.

The coefficient of friction (COF) is one of the most important parameters characterizing the tribological performance of materials [32]. Figure 4 shows the friction coefficients and their changing trends of polyurethane composite samples under loads of 0.5 and 0.7 MPa and at different rotational speeds. Figure 5 shows the average friction coefficient of the samples.

It can be found from Figure 4 that the friction coefficient curves of the three composite materials with added fibers fluctuate less than those of the pure PU without added fibers. This confirms the addition of fibers can change parameters such as the roughness and hardness of the friction surface of the composite materials, thereby affecting the friction performance. Meanwhile, when the fibers come into contact with the friction surface, they can absorb part of the energy through deformation, fracture, and friction, thereby reducing the energy transferred to the polyurethane matrix [33]. This energy dissipation mechanism can reduce the fluctuation of the friction coefficient and make the friction coefficient curve more stable [34].

Under most working conditions, the friction coefficient curve of ARF is always at the lowest position, and the curve is more stable compared with the other three composite materials. The friction coefficient of PPF is the largest, while that of UF and PU is similar. Moreover, the friction coefficient curves of these three materials all fluctuate greatly. Therefore, it can be known that in this experiment, the addition of aramid fibers significantly reduced the friction coefficient of PU and significantly improved the stability of the friction coefficient. However, the addition of polypropylene fibers had a negative impact on the friction coefficient of the PU matrix, while the addition of UHMWPE fibers had a relatively small impact on PU. This is related to multiple factors, such as the mechanical properties of the composite material and the inherent characteristics of the fibers. It is worth noting that in this test, the ARF material with the best comprehensive mechanical properties also has the lowest and most stable COF. It can be observed from Figure 4 that the introduction of aramid fibers reduces the interfacial shear strength of the composite and may induce a partial film-supporting effect. In combination with their inherent high hardness and strength, these ARF samples can more effectively impede deformation during friction, ultimately leading to a relatively lower COF. In addition, aramid fibers have a high crystallinity and a rigid molecular chain structure, which makes their surface relatively smooth and gives them a low COF. The benzene ring structure in their molecular chains may form a self-lubricating layer, reducing energy loss during the friction process [35,36].

Analysis of the average COF reveals that under the six working conditions of this test, the average COF of the ARF remains at the minimum value, the average COF of the PPF is the largest, and the average COF of the UF and PU are similar. This result is consistent with the real-time COF in Figure 4. After adding aramid fibers to the PU matrix, the average COF of the material decreased significantly. It was the lowest under the working condition of 0.5 MPa-250 r/min, which was 57.73% lower than that of PU under the same conditions, reaching 0.093. However, the addition of polypropylene fibers and UHMWPE fibers has a relatively small effect on reducing the COF of the PU. This may be related to the bonding situation between the fibers and the matrix as well as the state of the friction surface. The relevant contents are discussed in detail in the part about observing the surface microscopic morphology. Furthermore, under the same load conditions, the average COF of PU, UF, and PPF, which have relatively lower hardness and strength, all decrease with the increase in rotational speed. This confirms, on the one hand, when the rotational speed increases, the flow rate of distilled water increases, and the lubricating pressure between the two interfaces decreases, resulting in an internal and external lubricating pressure difference. This causes more distilled water to enter the gap, the proportion of zones with elastohydrodynamic lubrication increases, and the lubrication state to improve. Therefore, the COF of the sample decreases. On the other hand, at high rotational speeds, the temperature of the friction surface rises, which may lead to material softening or the formation of a lubricating film, thereby reducing the COF. This can be supported by the surface morphology of the worn sample. However, the COF of the ARF actually increases as the rotational speed increases. This indicates that aramid fibers maintain a high modulus even at high temperatures, providing stable mechanical support and limiting the expansion of the contact area [37]. As the speed increases, no thermal melting lubrication occurs at the interface. The friction is mainly dominated by the compacted third-body layer and the mechanical interlocking of the fiber protrusions. This can be confirmed by observing the wear surface of the ARF sample. Based on the above analysis, the addition of the three fibers has a positive impact on the stability of the COF of the fiber-wound composite material. Among them, ARF has the lowest and most stable coefficient of friction and shows the most excellent tribological performance during the test process.

### 3.4. Wear Mass Loss Analysis

**Figure 6:**
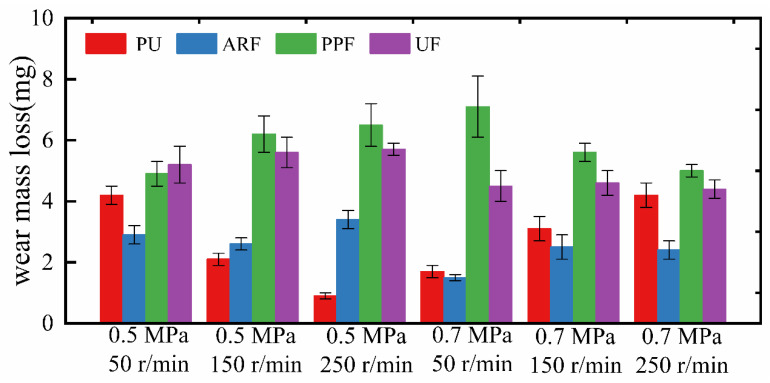
Wear mass loss of samples under different test conditions. (The error bars represent the standard deviation).

Wear mass loss can be used as one of the indicators for evaluating the wear resistance of materials. Figure 6 shows the wear quality of four different materials under six different working conditions.

In this study, for all the conditions, the wear results were reported by the mass loss after a fixed test duration (7200 s). However, considering that the sliding distance of the sample increases exponentially with different rotational speeds, inspired by the Archard-type wear analysis model [38], we introduced a specific wear rate K to standardize the sliding distance and the applied load, enabling a more rigorous comparison of wear behavior under different operating conditions. Calculate the wear rate K according to Equation (1):

(1) $$K=\frac{Dm}{Lsliding\cdot F}$$

where Dm is the wear mass loss for the sample, mg/(N·m); Lsliding is the sliding distance, m; and F is the applied load, N. Meanwhile, the sliding distance Lsliding and the applied load F are calculated according to Equations (2) and (3):

(2) $$Lsliding=\frac{\pi n\cdot (D1-D2)\cdot t}{60}$$

(3) $$F=\frac{({D1}^{2}-{D2}^{2})\pi \cdot P\cdot 10^{6}}{4}$$

where n represents the rotational speed, r/min; D1 represents the outer diameter of the sample, m; D2 represents the inner diameter of the sample, m; t stands for wear time, s; P stands for pressure, MPa.

Table 3 shows the wear rates of all the samples under different operating conditions.

It can be observed from Figure 6 and Table 3 that the wear mass loss of PU decreases after adding aramid fibers. Especially under the high-load test condition of 0.7 MPa, when the rotational speed is the same, the wear mass loss of ARF is all less than that of PU. Furthermore, the minimum and second-minimum wear rates of the ARF samples were achieved under a 0.7 MPa condition. Among them, under the working condition of 0.7 MPa-50 r/min, the wear mass of ARF is only 1.5 mg, which is 12% lower than that of PU under the same condition. Furthermore, under the working condition of 0.7 MPa-250 r/min, the wear rate of ARF is only 57% of that of PU. This indicates that the aramid fiber-reinforced polyurethane sample ARF can withstand harsher working conditions without significant wear. This is because the high hardness and strength of aramid fibers can effectively suppress the indentation on the grinding pair and resist the shear force and plastic deformation during the friction process. Meanwhile, the extremely low wear of aramid fibers also indicates that the aramid fibers have a good bond with the polyurethane matrix, and the fibers are not prone to debonding, which can effectively transfer the load.

However, the wear mass loss of UF and PPF is significantly greater than that of PU. This is because, on the one hand, the melting points of UHMWPE fibers and polypropylene fibers are relatively low. The frictional heat accumulated during the friction process will cause the fibers to become locally softened, resulting in the failure of fiber support and accelerating wear. On the other hand, PPF and UF exhibit poor mechanical properties. The lower strength and modulus can cause the fibers parallel to the friction direction on the sample surface to bend and break, thus losing the fiber reinforcement effect. When PPF and UF experience significant wear, it is considered that the failed fibers become the source of wear. During the continuous friction process, the fibers detach from the matrix or even peel off, accelerating the material loss. In this section, aramid fibers exhibit the best wear resistance due to their excellent mechanical properties and the superior characteristics of the fibers themselves.

### 3.5. Wear Surface Topography of the Specimens

**Figure 7:**
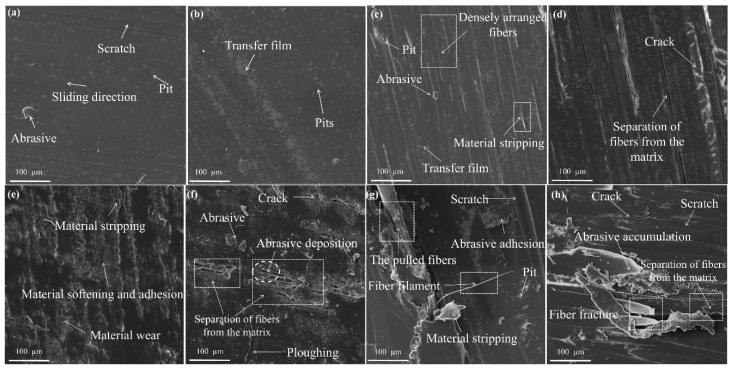
Surface micro-wear morphology of (**a**,**b**) PU and filament-winding composite materials (**c**,**d**) ARF, (**e**,**f**) UF, and (**g**,**h**) PPF.

In order to better study the influence of different fiber materials on the tribological performance of fiber-wound composites and describe the friction and wear conditions of the materials more accurately, the surface of the tested composites was observed by scanning electron microscopy (SEM). Based on previous studies, the actual maximum wear of water-lubricated bearings occurs under low-speed and heavy-load conditions [32]. Therefore, the SEM analysis mainly focuses on testing the morphology of the samples under a pressure of 0.7 MPa. Figure 7 shows the surface microstructure of PU and different filament-winding composite materials. Specifically, Figure 7a,b were taken from different locations of the same PU specimen, and the composite materials in this study were similarly sampled from different locations of their respective individual specimens.

As can be seen from Figure 7a, abrasive, uneven texture, scattered spots, and scratches appear on the surface of PU. In Figure 7b, discontinuous transfer films and a large number of pits can also be observed, indicating that there is abrasive wear and adhesive wear on the sample surface, and stress concentration occurs under high stress conditions, and pits are also produced as a result. It can be seen from Figure 7c,d that the fibers at most positions on the surface of ARF maintain a highly ordered one-dimensional dense arrangement structure, with only a small amount of local micrometer-level (<5 µm) debonding phenomenon, and the fibers do not undergo obvious plastic deformation, fracture, or pulping behavior. This phenomenon indicates that aramid fibers can effectively enhance the PU matrix through the load transfer effect, enabling the formation of composites with superior mechanical properties to withstand high loads. Meanwhile, a large number of discontinuous transfer films can be observed on the surface of ARF, which may be related to the benzene ring structure in the aramid molecular chain [39], which is conducive to reducing the coefficient of friction and the amount of wear. In addition, on the surface of ARF, a small number of abrasions and pits can be observed, as well as tiny cracks extending along the load direction. This is caused by stress concentration at certain points on the material surface exceeding the yield strength of the matrix. It also indicates that there is slight abrasive wear on the surface of ARF.

By observing Figure 7e, it can be found that, obviously, material softening and adhesion processes occur on the surface of UF. On the one hand, UHMWPE fibers have high toughness and are more prone to adhesion [40]. On the other hand, UHMWPE fibers have the lowest melting point among the fibers in this study. The melting points of UHMWPE and polypropylene fibers are around 130 °C and 160 °C, respectively; in contrast, oriented aramid fibers undergo thermal decomposition at approximately 560 °C. It is possible that the continuous accumulation of heat generated by friction leads to the thermal softening of part of the fibers. As a result, the fibers are more likely to deform and transfer, thereby increasing the possibility of adhesive wear. As the friction time increases, some adhesions transform into abrasives and ploughing, and cracks along the load direction also occur accordingly. Furthermore, as shown in Figure 7f, there is a distinct separation phenomenon at the interface between the matrix and the fibers on the UF surface. Although there is no obvious fiber breakage or being pulled out of the matrix, the detached polyurethane fragments are scattered all over the surface and form abrasive deposition between the fiber gaps. The surface of the sample is crisscrossed with grooves, which deteriorates the lubrication condition and intensifies the wear.

By observing Figure 7g, it can be found that there is a distinct separation phenomenon at the interface between the fibers and the matrix on the surface of PPF, and a large number of fibers are broken or removed from the polyurethane matrix. This is because the relatively low mechanical properties of polypropylene fibers cause them to be unable to resist the shear force under the cyclic friction of heavy loads and thus be pulled out of the matrix or damaged. The exposed fibers become a new source of wear, being worn and generating a large amount of abrasive and fiber fragments. A large amount of material on the surface also falls off, resulting in severe abrasive wear. The surface roughness of the sample is extremely high (Sa is approximately 6.4 μm, and the surface roughness parameters of other samples are discussed in detail in Section 3.6), and the friction coefficient and wear mass loss of the material sharply increase as a result. Furthermore, as shown in Figure 7h, a large number of parallel scratches and grooves were also found on the surface of PPF, accompanied by the characteristics of material softening.

### 3.6. Wear Morphologies of Tin Bronze Plates

**Figure 8:**
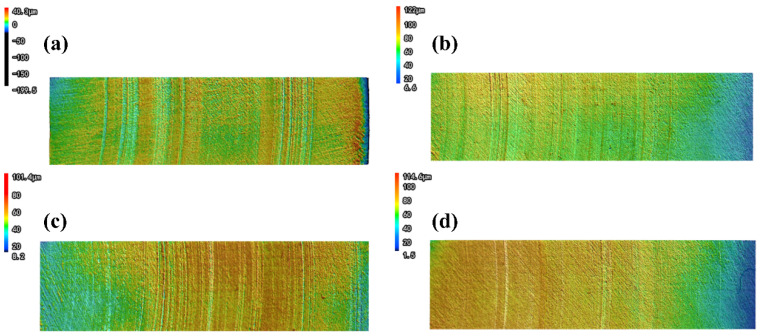
The surface wear morphology of the tin bronze plate under 0.7 MPa: (**a**) PU; (**b**) ARF; (**c**) UF; and (**d**) PPF.

**Figure 9:**
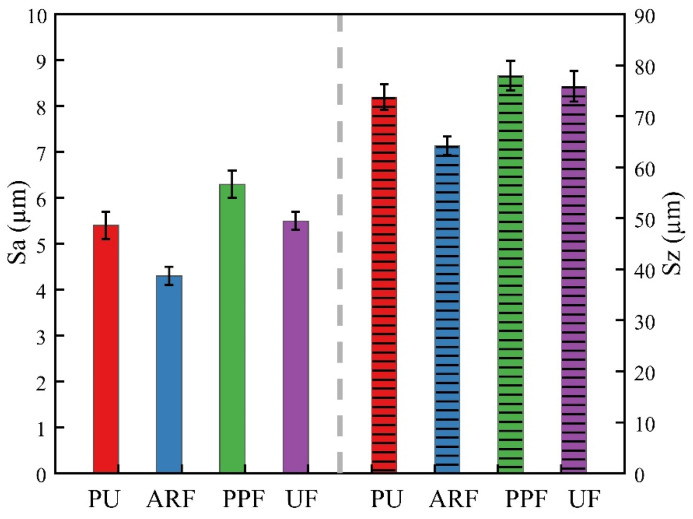
Surface characteristic parameters of tin bronze plate.

The friction pair of the water-lubricated bearing is composed of a metal drive shaft and a polymer bearing. Due to the complexity of the preparation process and the fine size requirements, it is necessary to analyze the wear morphology of the tin bronze plate used in the test. Tin bronze plates subjected to 0.7 MPa loading were selected for detailed examination, as this maximum pressure condition is presumed to generate the most severe wear patterns. In particular, when using the laser scanning confocal microscope, in order to more comprehensively characterize the wear condition of the surface of the tin bronze plates, a linear area that runs through the entire radius of the circular ring was selected, as shown in Figure 8. The parameters shown in Figure 9 were obtained by averaging the surface roughness values of multiple measurement areas.

From the wear morphology of the copper plate, it can be seen that the four materials show traces of wear to varying degrees along the direction of friction. The grinding marks on the surface of the tin bronze plate ground against PU vary in depth and are unevenly distributed, with the height difference of the roughness peaks being the most obvious. However, the grinding marks on the surfaces of the other three samples formed by fiber winding are more uniform. This is because the PU sample has local high-pressure points, heat-softening zones, and transfer film rupture under water-lubricated conditions, resulting in the randomness of the scratch depth and direction. In the specimens formed by fiber winding, the fibers are highly oriented and arranged, which improves the pressure distribution and thermal stability and makes the grinding marks tend to be uniform. It can be found that the grinding marks on the surface of the copper disk ground against ARF are the shallowest and most uniform. This indicates that the effect of enhancing the mechanical properties of aramid fibers and dispersing the load on the contact surface is the most obvious, which can effectively improve the smoothness of the sample surface and its tribological performance. Obvious material adhesion can be seen on the surface of the copper disk ground against UF, which indicates that the surface of the UF sample has undergone thermal softening and adhesive wear, consistent with the analysis in the previous text. A small amount of adhesion and slightly disordered scratches can be found on the surface of the copper plate ground against PPF.

In Figure 9, Sa represents the average roughness, and Sz represents the maximum peak–valley height. The average roughness and the maximum peak–valley height of the copper disks ground against PPF remained at the highest position. On the contrary, the average roughness and the maximum peak and valley heights of the copper disks ground against the ARF specimens remained at the lowest positions. In addition, the average roughness and maximum peak–valley height of the copper disks ground against the UF samples are similar to those of the PU samples, and this result is consistent with the average friction coefficient of the samples. ARF can effectively enhance the mechanical properties of materials, improve the lubrication conditions of the contact surface, and reduce the surface roughness of the specimens. UHMWPE fibers also have relatively good mechanical properties and lubricity. However, the surface of the UF samples underwent thermal softening and adhesive wear. The deformed fibers and resin matrix adhered to the surface of the copper disk, significantly increasing the surface roughness of the samples and the height difference of the surface roughness peaks. Due to its poor strength and rigidity, the polypropylene fibers of PPF are cut or pulled out from the matrix by large shear stress. The broken or pulled fibers are further worn, resulting in a further increase in the surface roughness of the sample and a significant reduction in surface flatness. The test results are consistent with the surface wear morphology images of the corresponding composite material samples.

### 3.7. Wear Mechanism

**Figure 10:**
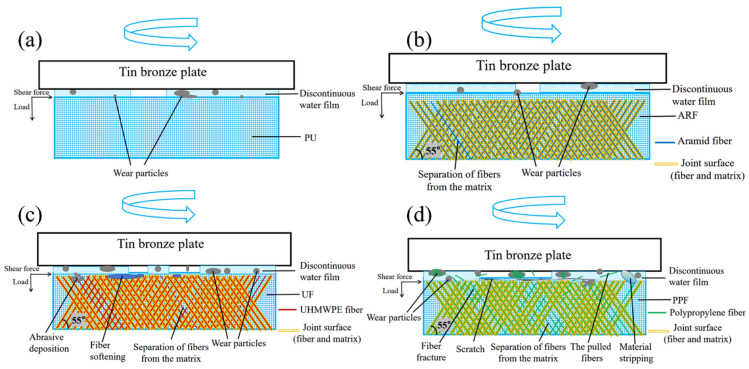
Schematic diagram of the wear mechanism of fiber-wound composites: (**a**) PU; (**b**) ARF; (**c**) UF; and (**d**) PPF.

The schematic diagrams of the wear mechanisms of different fiber-wound composite materials are shown in Figure 10. When studying the influence of fiber materials on the tribological performance of fiber-wound composites, based on considering the differences in the properties of three different fibers, namely aramid fibers, UHMWPE fibers, and polypropylene fibers, combined with the test results of friction coefficient, wear quality, and wear surface morphology in this study, an in-depth analysis of the wear mechanism was conducted.

As shown in Figure 10, the cross-linked structure of PU makes the material less likely to undergo plastic deformation under force. Therefore, the surface damage after friction is not obvious, the surface is relatively flat, and only slight abrasive wear occurs. During the friction process, only slight fiber separation from the matrix occurs in ARF (at the micrometer level). Meanwhile, according to the test data, the modulus, tensile strength, and bending strength of ARF are much higher than those of PU. Therefore, during the friction process, ARF can effectively bear most of the load, generate a partial film support effect, and more efficiently reduce the deformation of the composite material surface, thereby reducing wear. Furthermore, no obvious severe wear marks were observed on the surfaces of the tin bronze disk and the composite sample, which proves that the lubrication state of the ARF sample surface is relatively good. Therefore, the ARF sample is likely to be in a mixed lubrication state, with a regional dynamic discontinuous water film existing between the contact surfaces of the friction pair.

The tribological performance of UF in this test was similar to that of PU. Although UHMWPE fibers have a relatively low coefficient of friction due to their low surface energy and soft viscoelastic characteristics [41], under the harsh working conditions of low speed and heavy load, the accumulation of extremely large shear stress and frictional heat causes adhesion on the surface of UF samples, as shown in Figure 10c. Meanwhile, under extreme test conditions, a small portion of the fibers and the matrix undergo debonding. After debonding, microcracks are generated between the fibers and the matrix, increasing the wear particles and further intensifying the wear. The low friction characteristics of UHMWPE fibers are masked by interfacial debonding and the adhesion effect on the sample surface. Instead, their low modulus and high toughness lead to the deterioration of the overall tribological performance of the material.

The tribological performance of PPF is the poorest. PPF specimens with low hardness and strength cannot support heavy loads, causing the fibers to be cut off by the shear stress during the friction process or even pulled out from the resin matrix. The surface of the specimen, the fibers inside the specimen, and the matrix are all severely damaged, resulting in a large amount of material spalling, as shown in Figure 10d. Meanwhile, the exposed fibers and the fibers pulled out from the matrix are continuously worn between the contact surfaces of the friction pair, significantly increasing the surface roughness and intensifying the wear between the sample and the tin bronze plate.

## 4. Conclusions

In this study, by comparing the friction and wear properties of three different filament-winding composite materials (ARF, UF, PPF) in the water-lubricated environment, the influence of fiber materials on them was analyzed and discussed. The conclusions drawn from this investigation are shown as follows:

(1) ARF outperformed PU in tensile strength, flexural strength, and hardness, exhibiting a 3.7-fold increase in tensile strength and a 14.4% enhancement in hardness. Although PP and UHMWPE fibers improved the tensile properties of the composites, they displayed distinct performance profiles in compression, flexure, and hardness.
(2) Under the designated working conditions, ARF exhibited the lowest and most stable coefficient of friction (COF). Specifically, at 0.5 MPa and 250 r/min, the COF reached a minimum of 0.093, representing a 57.73% reduction compared to PU. Similarly, at 0.7 MPa and 50 r/min, the wear mass loss of ARF was 1.5 mg, a 12% decrease relative to PU. While the incorporation of aramid fibers effectively minimized friction and wear, the addition of UHMWPE fibers yielded only marginal improvements in tribological performance. Conversely, the inclusion of polypropylene fibers led to a sharp increase in both friction coefficient and wear mass loss.
(3) Worn surface morphology analysis revealed that ARF exhibited a dense fiber arrangement and superior surface flatness. The presence of minimal abrasive debris indicates that the fibers effectively reinforced the PU matrix, resulting in optimal tribological performance. In contrast, UF displayed distinct signs of adhesive wear accompanied by fiber–matrix debonding, which compromised its overall wear resistance. Finally, PPF demonstrated the poorest performance, characterized by severe failure mechanisms including fiber fracture, fiber pull-out, and extensive abrasive grains and wear tracks.

## Figures and Tables

**Table 1:** Qsn7-0.2 mechanical properties of tin bronze plates.

	Material	Yield Strength (MPa)	Tensile Strength (MPa)	Hardness (HB)
Tin bronze plates	Qsn7-0.2	≥170	≥355	≥70

**Table 2:** The average volume changes in samples for different soaking times.

Sample	PU	ARF	PPF	UF
Volume growth after 12 h	0.13%	0.32%	1.09%	0.56%
Volume growth after 24 h	0.97%	1.06%	1.99%	1.41%
Volume growth after 7 d	1.41%	1.47%	2.70%	1.91%

**Table 3:** The wear rate of the samples under different conditions.

Applied Load	Speed	PU	ARF	PPF	UF
226 N (0.5 MPa)	50 r/min	8.21 × 10^−5^	5.67 × 10^−5^	9.58 × 10^−5^	10.16 × 10^−5^
	150 r/min	1.37 × 10^−5^	1.69 × 10^−5^	4.04 × 10^−5^	3.65 × 10^−5^
	250 r/min	3.52 × 10^−6^	1.33 × 10^−5^	2.54 × 10^−5^	2.23 × 10^−5^
317 N (0.7 MPa)	50 r/min	2.37 × 10^−5^	2.09 × 10^−5^	9.91 × 10^−5^	6.28 × 10^−5^
	150 r/min	1.44 × 10^−5^	1.16 × 10^−5^	2.61 × 10^−5^	2.14 × 10^−5^
	250 r/min	1.17 × 10^−5^	6.70 × 10^−6^	1.40 × 10^−5^	1.23 × 10^−5^

## Data Availability

The raw data supporting the conclusions of this article will be made available by the authors on request.
